# Supplementary material for: Mitochondrial Genomes of Two Asexual Trichogramma (Hymenoptera: Trichogrammatidae) Strains and Comparison with Their Sexual Relatives
Source: Insects. 2022 Jun 16;13(6):549. doi: 10.3390/insects13060549 (PMC9224637; doi:10.3390/insects13060549)
Supplement: Supplementary file 1 [file insects-13-00549-s001.zip › insects-1756291-supplementary.pdf]

Supplemental Materials for

Mitochondrial genomes of two asexual *Trichogramma* (Hymenoptera: Trichogrammatidae) strains and comparison with their sexual relatives

This supplemental file includes

Table S1. Primers used in this study
Table S2. Statistics of Trichogramma mitochondrial genomes and their control regions
Table S3. Relaxed selection test of *T. cacoeciae* or *T. pretiosum* branch comparing to sexual branches
Figure S1. Secondary structures of tRNAs in *T. cacoeciae* mitochondrial genome
Figure S2. Secondary structures of tRNAs in *T. pretiosum* mitochondrial genome

Table S1. Primers used in this study

Primer	Sequence (5'-3')	Usage
**FP1**	GGAGGATTTGGAAATTGATTAGTTCC	For amplification of COX1 from *T.cacoeciae* and *T. pretiosum*
**RP1**	CCCGGTAAAATTAAAATATAAACTTC	
**FP2**	TTTCTTGAGCAATGTTATTAACAGC	For amplification of near full-length mitochondrial genome from *T.cacoeciae*
**RP2**	AAATCTACTGAAGGTCCCCTATGAG	
**FP3**	ATCTCATAGGGGTCCTTCTGTAGATTTATCAAT	For amplification of near full-length mitochondrial genome from *T. pretiosum*
**RP3**	AAGATAATGGAGGATACACAGTTCAACCTGTTC	
**FP4**	TTGATATTGACATTTTGTGGATGTAGTT	For closing the gap between COX1 and COX3 in *T. pretiosum* mitochondrial genome assembly
**RP4**	TTGAATTATGAATCCAATGACTTAAACT	
**FP5**	ATTACTGTAGCTCCCCAAA	For amplification of nad4l from *T. pretiosum*
**RP5**	AGGAATCAACCTTATCTCAAA	

FP: forward primer; RP: reverse primer.

Table S2. Statistics of Trichogramma mitochondrial genomes and their control regions

Species	Mitochondrial genome		Control region	
	Length	AT%	Length	AT%
T. cacoeciae	16034	84.9	756	87.6
T. pretiosum	16227	85.2	669	91.9
T. chilonis	16147	85.2	668	89.2
T. ostriniae	16,472	85.4	830	89
T. dendrolimi	16878	84.8	1320	88
T. japonicun	15962	84.9	595	91.1

Table S3. Relaxed selection test of *T. cacoeciae* or *T. pretiosum* branch comparing to sexual branches

Gene	*T. cacoeciae*				*T. pretiosum*			
	K	LR	*p*	*q*	K	LR	*p*	*q*
**atp6**	0.74	0.49	0.484	0.860	1.88	3.03	0.082	0.383
**atp8**	33.99	0.89	0.346	0.860	0.70	0.51	0.477	0.742
**cob**	0.88	0.29	0.592	0.860	1.15	0.35	0.552	0.742
**cox1**	1.00	$-4.4e^{-5}$	1.000	1.000	1.07	0.09	0.759	0.759
**cox2**	1.51	0.99	0.319	0.860	1.82	1.12	0.291	0.742
**cox3**	1.17	0.18	0.676	0.860	0.93	0.11	0.737	0.759
**nad1**	1.27	0.44	0.505	0.860	2.65	11.57	0.001	0.007
**nad2**	1.04	0.01	0.924	1.000	1.26	0.70	0.404	0.742
**nad3**	1.23	0.22	0.640	0.860	20.41	0.48	0.488	0.742
**nad4**	1.16	0.26	0.608	0.860	5.67	132.56	0	0
**nad4l**	0.93	0.00	1.000	1.000	15.86	0.16	0.688	0.759
**nad5**	0.75	2.55	0.110	0.770	0.71	2.48	0.115	0.403
**nad6**	0.82	0.26	0.614	0.860	0.85	0.30	0.583	0.742
**13PCGs**	0.90	2.72	0.099	0.770	1.19	0.79	0.375	0.742

Terminal branches of *T. cacoeciae* or *T. pretiosum* was set as the test, separately. Other terminal and internal branches were set as the reference. A K value significantly less than 1 indicates relaxed selection. *P* values were corrected for multiple tests using Benjamini and Hochberg method. K: relaxation or intensification parameter; LR: likelihood ratio; 13PCGs: concatenated 13 mitochondrial protein-coding genes.

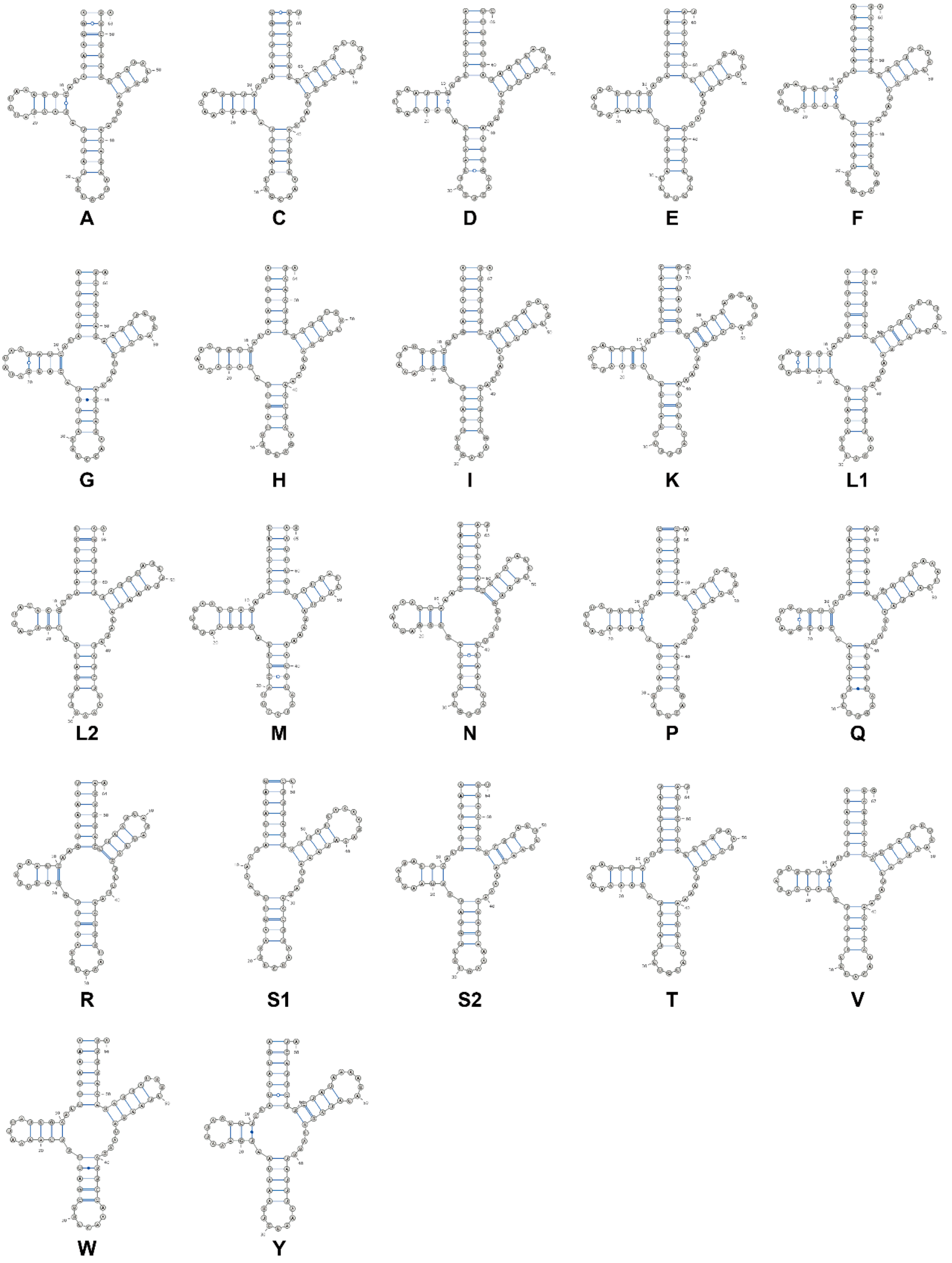

Figure S1. Secondary structures of tRNAs in *T. cacoeciae* mitochondrial genome.

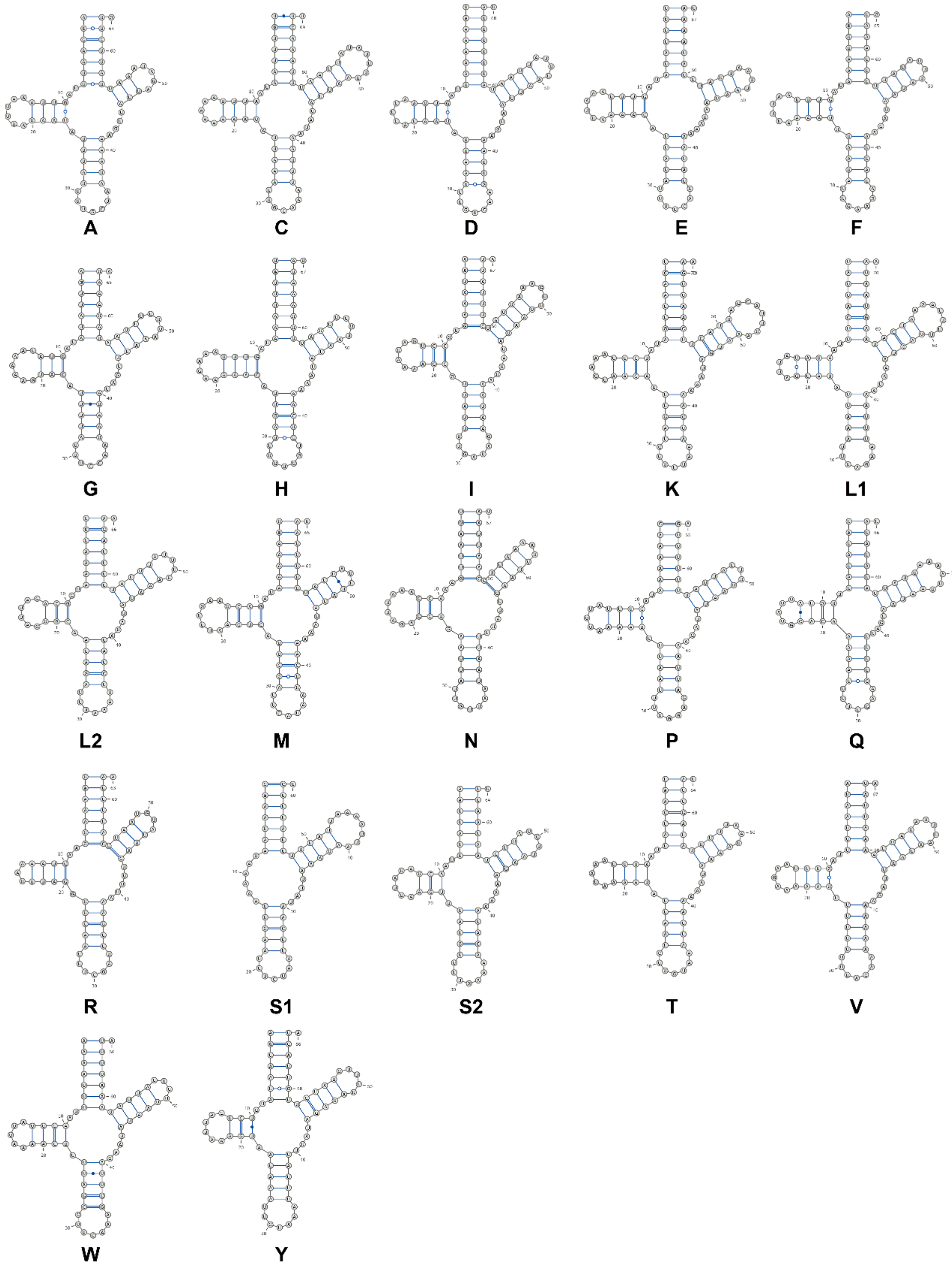

Figure S2. Secondary structures of tRNAs in *T. pretiosum* mitochondrial genome.
